# Isolation and Cytotoxicity Evaluation of the Chemical Constituents from *Cephalantheropsis gracilis*

**DOI:** 10.3390/ijms16023980

**Published:** 2015-02-12

**Authors:** Chi-Fen Chang, Yu-Lin Hsu, Chao-Ying Lee, Chia-Hua Wu, Yang-Chang Wu, Ta-Hsien Chuang

**Affiliations:** 1 Department of Anatomy, School of Medicine, China Medical University, Taichung 40402, Taiwan; E-Mail: cfchang@mail.cmu.edu.tw; 2 Department of Chemistry, National Cheng Kung University, Tainan 70101, Taiwan; E-Mail: lynn4051@yahoo.com.tw; 3 School of Pharmacy, China Medical University, Taichung 40402, Taiwan; E-Mails: cylee@mail.cmu.edu.tw (C.-Y.L.); u101003312@cmu.edu.tw (C.-H.W.); 4 Research Center for Chinese Herbal Medicine, China Medical University, Taichung 40402, Taiwan; 5 Chinese Medicine Research and Development Center, China Medical University Hospital, Taichung 40402, Taiwan; 6 Graduate Institute of Natural Products, Kaohsiung Medical University, Kaohsiung 80708, Taiwan; 7 Center of Molecular Medicine, China Medical University Hospital, Taichung 40402, Taiwan

**Keywords:** *Cephalantheropsis gracilis*, Orchidaceae, quinazoline, tryptanthrin, indolotryptanthrin, dihydrophenanthrene, cytotoxicity

## Abstract

*Cephalantheropsis gracilis* afforded five new compounds: cephalanthrin-A (**1**), cephalanthrin-B (**2**), cephathrene-A (**3**), cephathrene-B (**4**), methyl 2-(aminocarbonyl)phenylcarbamate (**5**), and 52 known compounds. The structures of the new compounds were determined by spectroscopic analysis. Among the compounds isolated, tryptanthrin (**6**), phaitanthrin A (**7**), cephalinone D (**19**), and flavanthrin (**30**) showed significant cytotoxicity against MCF-7, NCI-H460, and SF-268 cell lines.

## 1. Introduction

**Figure 1:**
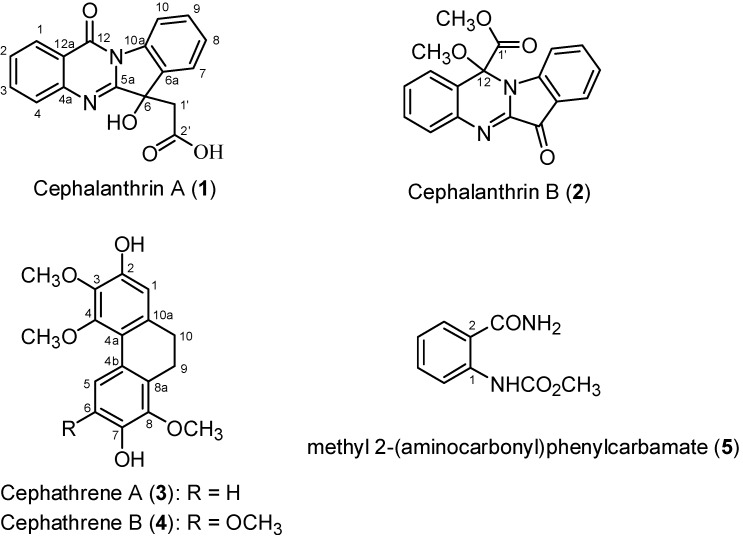
Structures of five new compounds **1**–**5**.

The genus *Cephalantheropsis* (also known as *Cephalanceropsis*) belongs to the Orchidaceae family and is comprised of eight species distributed in Southeast Asia. The plant, *Cephalantheropsis gracilis* (Lindl.) Shiu-Ying Hu var. gracilis, is an orchid native to Taiwan and grows in forests at altitudes of 500–1500 m throughout the island [1]. The crude methanol extract of *C. gracilis* showed significant cytotoxicity against human breast carcinoma (MCF-7), lung carcinoma (NCI-H460), and central nervous system carcinoma (SF-268) cell lines in our preliminary screening. In earlier papers, the isolation of indole alkaloids was reported from *C. gracilis*, but they are unlikely to be responsible for such anticancer activity [2,3]. In the course of continuing the search for bioactive molecules from *C. gracilis*, two new quinazolines, cephalanthrin-A (**1**) and cephalanthrin-B (**2**), two new dihydrophenanthrenes, cephathrene-A (**3**) and cephathrene-B (**4**), and a methyl 2-(aminocarbonyl)phenylcarbamate (**5**) [4] (Figure 1) as well as 52 known compounds were obtained and identified from a methanol extract (in addition to common long-chain fatty acids, chlorophylls, and steroids). Herein, we describe the structural elucidation of these new compounds and the cytotoxic properties of all compounds identified toward several human cancer cell lines.

## 2. Results and Discussion

Cephalanthrin-A (**1**) was isolated as an optically active white amorphous powder. The molecular formula was determined to be C_17_H_12_N_2_O_4_ from a molecular ion of *m*/*z* 308.0794 by HREIMS. In the IR spectrum, a very broad band at 3000 cm^−1^ and an absorption band at 1652 cm^−1^ both indicated the presence of a carboxylic acid. The 1D ^1^H and ^13^C NMR data (Table 1, Figure S1), together with 2D COSY, HMQC, and HMBC spectra, revealed two sets of *o*-disubstituted benzene rings, one at δ_H_ 7.59 (t, *J* = 7.5 Hz, H-2), 7.77 (d, *J* = 7.5 Hz, H-4), 7.83 (t, *J* = 7.5 Hz, H-3), and 8.34 (1H, d, *J* = 7.5 Hz, H-1); δ_C_ 123.0 (C-12a), 127.4 (C-1), 128.0 (C-2), 128.5 (C-4), 135.2 (C-3), and 148.4 (C-4a). The other was at δ_H_ 7.38 (t, *J* = 7.7 Hz, H-8), 7.51 (t, *J* = 7.7 Hz, H-9), 7.73 (1H, d, *J* = 7.7 Hz, H-7), and 8.49 (1H, d, *J* = 7.7 Hz, H-10); δ_C_ 117.2 (C-10), 124.6 (C-7), 127.3 (C-8), 130.8 (C-9), 134.2 (C-6a), and 140.8 (C-10a). The ^13^C NMR data also indicated the presence of an imino C-5a (δ 161.9) and an amidic C-12 (δ 160.0). These signals appear to be very closely related to indolo[2,1-*b*]quinazoline-6,12-dione (tryptanthrin, **6**), except that the carbonyl C-6 is replaced by a saturated quaternary carbon (δ 75.9). NMR spectroscopic data also allowed us to determine the remaining two substituents on C-6 being an –OH group (δ 3.81) and a –CH_2_COOH group (δ_H_ 3.45, 3.55 (each 1H, d, *J* = 16.5 Hz, H-1'); δ_C_ 43.6 (C-1') and 170.9 (C-2')). The methylene protons adjacent to a quaternary carbon (C-6) could be split due to either chirality or steric hindrance. HMBC correlations from H-1 to C-12, H-7 to C-6 and H-1' to C-5a, C-6 and C-6a, as well as the NOE correlations between H-1' and H-7, supported a structure of 6-hydroxy-6-(carboxymethyl)-tryptanthrin for cephalanthrin-A (**1**).

**Table 1:** ^1^H and ^13^C NMR Spectroscopic Data and HMBC Correlations for Cephalanthrins **1** and **2**.

Position	1 in Acetone-*d*_6_ (300 MHz/75 MHz)			2 in CDCl_3_ (300 MHz/75 MHz)		
	δ_H_ (*J* in Hz)	δ_C_	HMBC	δ_H_ (*J* in Hz)	δ_C_	HMBC
1	8.34 d (7.5)	127.4	C-3, -4a, -12	7.60 d (7.6)	126.1	C-3, -4a, -12
2	7.59 t (7.5)	128.0	C-4, -12a	7.41 t (7.6)	129.3	C-4, -12a
3	7.83 t (7.5)	135.2	C-1, -4a	7.52 t (7.6)	131.4	C-1, -4a
4	7.77 d (7.5)	128.5	C-2, -12a	7.72 d (7.6)	129.7	C-2, -12a
4a		148.4			141.9	
5a		161.9			144.7	
6		75.9			184.0	
6a		134.2			120.5	
7	7.73 d (7.7)	124.6	C-6, -9, -10a	7.83 d (7.6)	125.5	C-6, -9, -10a
8	7.38 t (7.7)	127.3	C-6a, -10	7.20 t (7.6)	124.0	C-6a, -10
9	7.51 t (7.7)	130.8	C-7, -10a	7.59 t (7.6)	137.9	C-7, -10a
10	8.49 d (7.7)	117.2	C-6a, -8	7.23 d (7.6)	112.3	C-6a, -8
10a		140.8			148.9	
12		160.0			87.4	
12a		123.0			120.5	
1'	3.45 d (16.5) 3.55 d (16.5)	43.6	C-5a, -6, -6a, -2'		167.8	
2'		170.9				
6-OH	3.81 s					
1'-OCH_3_				3.71 s	53.8	C-1'
12-OCH_3_				3.07 s	50.6	C-12

Cephalanthrin-B (**2**), also isolated as an optically active yellow amorphous powder, was determined to have a molecular formula of C_18_H_14_N_2_O_4_. By comparison of the ^1^H and ^13^C NMR spectra of **2** (Figure S2) with those of tryptanthrin (**6**) and cephalanthrin-A (**1**), an oxygenated quaternary carbon (δ 87.4) was shown to replace the carbonyl C-12 to form a 12,12-disubstituted tryptanthrin (Table 1). Methoxyl (δ_H_ 3.07; δ_C_ 50.6) and methoxycarbonyl (δ_H_ 3.71; δ_C_ 53.8 and 167.8) substituents were also identified, and their presence was confirmed by HMBC correlations from both H-1 and 12-OCH_3_ to C-12 and the NOE correlations between H-1, H-10 and 1'-OCH_3_, 12-OCH_3_. Hence, a structure of 12-methoxy-12-(methoxycarbonyl)-tryptanthrin was deduced for cephalanthrin-B (**2**). Although compound **2** has been synthesized by Cornforth* et al.* [5], this represents the first isolation of a pure compound from a natural source.

Due to the small specific rotations of compounds **1** ([α]_D_ +8.0°) and **2** ([α]_D_ +3.0°), we suspected they might be not optically pure compounds. The configuration of compounds **1** and **2** has not been determined due to the isolation of insufficient amounts of these materials. However, we adopted the similar structure of phaitanthrin A (**7**) as a model. First, our attempts to synthesize a pair of diastereomeric esters by acylating **7** with (+)-α-methoxy-α-trifluoromethylphenylacetyl chloride [(+)-MTPACl] [6], even with acetyl chloride, were unsuccessful. This most likely is due to steric inhibition at the tertiary alcohol, which is present in the tryptanthrin skeleton. We then tried to analyze the C-6 chemical shift behaviors using the chiral shift reagents, tris[3-trifluoroacetyl-d- and l-camphorato]europium(III) [(*R*)- and (*S*)-Eu(tfc)_3_] [7], again to no avail.

Cephathrene-A (**3**) was isolated as a white amorphous powder with a molecular formula of C_17_H_18_O_5_ as determined by the molecular ion peak at *m*/*z* 302.1153 in HREIMS data. UV absorptions at 267 and 304 nm indicated the presence of a benzene system, and the IR spectrum revealed an OH absorption band at 3404 cm^−1^. From the ^1^H NMR spectrum, two mutually-coupled aromatic protons at δ 6.86 (1H, d, *J* = 8.7 Hz, H-6) and 7.93 (1H, d, *J* = 8.7 Hz, H-5) and a proton at δ 6.63 (1H, s, H-1) suggested the presence of 1,2,3,4-tetrasubstituted and pentasubstituted benzene rings, respectively (Table 2, Figure S3). We also found two mutually coupled aliphatic signals at δ 2.66 (2H, m, H-10) and 2.79 (2H, m, H-9), which were assigned to an ethylene group. HMBC correlations of H-9 with C-4b, C-8, C-8a, C-10, and C-10a, as well as H-10 with C-1, C-4a, C-8a, C-9, and C-10a indicated that the two benzene rings are linked together by the ethylene group. Furthermore, the HMBC correlation between H-5 and C-4a established the existence of a bond between C-4a and C-4b. Thus, compound **3** possessed a 9,10-dihydrophenanthrene skeleton. The HMBC correlations of H-1, H-5, H-6, H-9, and H-10 allowed for the identification of the quaternary aromatic carbons, C-2, C-3, C-4a, C-4b, C-7, C-8, C-8a, and C-10a. We determined the identity of five other substituents, two hydroxyls and three methoxyls. The hydroxyl signal at δ 5.65 showed HMBC correlations with C-6, C-8, and C-7, and the other hydroxyl signal at δ 5.70 showed HMBC correlations with C-1, C-3, and C-2, indicating that the two hydroxyl groups are attached to C-7 and C-2, respectively. Whereas the three methoxyl signals at δ 3.75, 3.79, and 3.96 were shown to be located at C-4, C-8, and C-3, respectively, owing to the HMBC correlations of 3-OCH_3_ with C-3, 4-OCH_3_ with C-4, and 8-OCH_3_ with C-8. Additional evidence for the positions of these substituents came from the NOE correlations between 2-OH and H-1, 3-OCH_3_; H-5 and 4-OCH_3_; H-1 and H-10; 8-OCH_3_ and 7-OH, H-9. Therefore, cephathrene-A (**3**) was assigned the structure 2,7-dihydroxy-3,4,8-trimethoxy-9,10-dihydrophenanthrene.

Cephathrene-B (**4**) was isolated as a white amorphous powder. The HREIMS showed a molecular ion consistent with the molecular formula C_18_H_20_O_6_. The spectral data showed a resemblance to compound **3** (Table 2). The ^1^H NMR spectrum (Figure S4) disclosed the presence of two singlet aromatic protons at 6.63 (H-1) and 7.75 (H-5), indicating a hexasubstituted 9,10-dihydrophenanthrene. The regiochemistries of the substituents, two hydroxyls and four methoxyls, were determined by HMQC, HMBC, and NOESY experiments. As in the case of **3**, the two hydroxyls at δ 5.59 and 5.70 are located at C-7 and C-2, respectively, whereas three of the four methoxyls at δ 3.75, 3.84, and 3.97 are at C-4, C-8, and C-3, respectively. The remaining methoxyl at δ 3.93 was thought to be at C-6 due to NOE correlations between H-5 and 4- and 6-OCH_3_. Thus, the structure of 2,7-dihydroxy-3,4,6,8-tetramethoxy-9,10-dihydrophenanthrene was established for cephathrene-B (**4**).

**Table 2:** ^1^H and ^13^C NMR Spectroscopic Data and HMBC Correlations for Cephathrenes **3** and **4**.

Position	3 in CDCl_3_ (400 MHz/100 MHz)			4 in CDCl_3_ (300 MHz/75 MHz)		
	δ_H_ (*J* in Hz)	δ_C_	HMBC	δ_H_ (*J* in Hz)	δ_C_	HMBC
1	6.63 s	109.9	C-2, -3, -4a, -10	6.63 s	110.1	C-2, -3, -4a, -10
2		147.6			147.7	
3		139.0			139.0	
4		150.5			150.4	
4a		120.3			120.3	
4b		125.9			124.2 *	
5	7.93 d (8.7)	124.2	C-4a, -7, -8a	7.75 s	106.6	C-4a, -4b, -6, -7, -8a
6	6.86 d (8.7)	112.8	C-4b, -8		145.5	
7		147.1			137.1	
8		143.6			143.7	
8a		130.8			124.3 *	
9	2.79 m	22.4	C-4b, -8, -8a, -10, -10a	2.73 m	21.5	C-4b, -8, -10, -10a
10	2.66 m	29.5	C-1, -4a, -8a, -9, -10a	2.64 m	29.8	C-1, -4a, -8a, -9
10a		134.5			135.2	
2-OH	5.70 s		C-1, -2, -3	5.70 s		
3-OCH_3_	3.96 s	61.1	C-3	3.97 s	61.2	C-3
4-OCH_3_	3.75 s	60.1	C-4	3.75 s	60.1	C-4
6-OCH_3_				3.93 s	56.3	C-6
7-OH	5.65 s		C-6, -7, -8	5.59 s		
8-OCH_3_	3.79 s	61.3	C-8	3.84 s	60.8	C-8

* Assignments may be interchangeable.

Compound **5**, with molecular formula C_9_H_10_N_2_O_3_, was isolated as a white amorphous powder. It had UV absorptions at 257, 289, and 311 nm, indicative of an aromatic system. The ^1^H NMR spectrum (Figure S5) showed resonances at δ 7.05 (1H, t, *J* = 7.8 Hz, H-4), 7.49 (1H, t, *J* = 7.8 Hz, H-5), 7.83 (1H, d, *J* = 7.8 Hz, H-3), and 8.38 (1H, d, *J* = 7.8 Hz, H-6) for an *o*-disubstituted benzene. The HMBC correlations from H-3 to an amide carbon (δ 172.0) and from OCH_3_ (δ 3.71) to a carbamate carbon (δ 154.6) and the NOE correlation between H-6 and an amine proton (δ 11.29) suggested the structure of methyl 2-(aminocarbonyl)phenylcarbamate for **5**. The ^1^H and ^13^C NMR spectra of **5** and the acylation product of *o*-aminobenzamide with methyl chloroformate (ClCO_2_CH_3_) were identical and further confirmed the structure of **5** (Equation (1)).


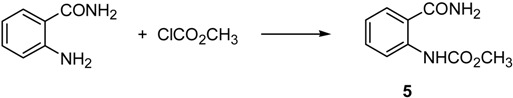
(1)

Other known compounds were also isolated from *C. grilis* including 7 quinazolines, tryptanthrin (**6**) [8], phaitanthrin A (**7**) [8], phaitanthrin B (**8**) [8], methylisatoid (**9**) [8], candidine (**10**) [8], 1*H*-quinazoline-2,4-dione (**11**) [9], and 3-(2-hydroxy-phenyl)-3*H*-quinazolin-4-one (**12**) [10]; 15 indole alkaloids, cephalandole A (**13**) [2], cephalandole B (**14**) [2], cephalandole C (**15**) [2], cephalinone A (**16**) [2], cephalinone B (**17**) [2], cephalinone C (**18**) [2], cephalinone D (**19**) [2], (*S*)-3-(2-oxopropyl)-3-hydroxyindolin-2-one (**20**) [2], methyl dioxindole-3-acetate (**21**) [2], isatan (**22**) [2], indigo (**23**) [2], indirubin (**24**) [2], isatin (**25**) [2], indole-3-carbaldehyde (**26**) [2], and indole-3-carboxylic acid (**27**) [2]; 2 indolotryptanthrins cephathrindole A (**28**) [3] and cephathrindole B (**29**) [3]; 5 dihydrophenanthrenes, flavanthrin (**30**) [11], coelonin (**31**) [11], 6-*O*-methylcoelonin (**32**) [12], 2,7-dihydroxy-3,4-dimethoxy-9,10-dihydrophenanthrene (**33**) [13], and 2,7-dihydroxy-3,4,6-trimethoxy-9,10-dihydrophenanthrene (**34**) [11]; 1 lignin, secoisolariciresinol (**35**) [14]; 1 flavonoid, kaempferol 3-rutinoside (**36**) [15]; 1 ionol, blumenol A (**37**) [16]; and 20 benzenoids, 2-aminobenzoic acid (**38**), methyl 2-aminobenzoate (**39**),* N*-cinnamoyltyramine (**40**),* N*-*p*-coumaroyltyramine (**41**),* N*-*trans*-feruloyltyramine (**42**), dihydroconiferyl dihydro-*p*-coumarate (**43**), 4-hydroxybenzaldehyde (**44**), 1-(4-hydroxy-phenyl)ethanone (**45**), 4-hydroxy-phenethyl alcohol (**46**), 3-(4-hydroxy-phenyl)-propionic acid (**47**), vanillin (**48**), vanillic acid (**49**), 4-hydroxy-3-methoxybenzyl alcohol (**50**), syringaldehyde (**51**), 3,5-dimethyl-4-hydroxypropiophenone (**52**),* trans*-*p*-coumaric acid (**53**), *trans*-ferulic acid (**54**), *cis*-ferulic acid (**55**), methyl *trans*-4-hydroxy-3-methoxycinnamate (**56**), and methylsinapat (**57**).

All the isolated compounds were subjected to cytotoxic evaluation against MCF-7, NCI-H460, and SF-268 cell lines. Tryptanthrin (**6**), phaitanthrin A (**7**), cephalinone D (**19**), and flavanthrin (**30**) showed significant cytotoxicity against MCF-7, NCI-H460, and SF-268 cell lines with IC_50_ values of 7.6–42.9 μM (Table 3).

**Table 3:** Cytotoxicity of active compounds toward three cancer lines.

Compound	IC_50_ (μM)		
	MCF-7	NCI-H460	SF-268
Tryptanthrin (**6**)	9.4 ± 0.3	8.5 ± 0.8	22.6 ± 1.1
Phaitanthrin A (**7**)	17.8 ± 0.8	17.3 ± 1.2	42.9 ± 1.0
Cephalinone D (**19**)	7.6 ± 0.7	7.8 ± 1.0	12.2 ± 1.3
Flavanthrin (**30**)	21.9 ± 1.5	22.8 ± 2.3	23.0 ± 2.0

Values were mean ± SD (*n* = 3–8); MCF-7 = human breast tumor cell line; NCI-H460 = human lung tumor cell line; SF-268 = human entral nervous system tumor cell line.

## 3. Experimental Section

### 3.1. General

Optical rotations were measured on a Jasco DIP-370 digital polarimeter (JASCO, Tokyo, Japan). UV spectra were recorded on an Agilent 8453 spectrophotometer (Agilent Technologies, Palo Alto, CA, USA). IR spectra were recorded on a Nicolet Magna FT-IR spectrophotometer (Nicolet Instrument, Inc., Madison, WI, USA). NMR spectra were recorded on a Bruker Avance 300 (Bruker, Karlsruhe, Germany) and AMX 400 spectrometers (Bruker, Karlsruhe, Germany), and all chemical shifts are given in ppm using tetramethylsilane (δ 0.00) as an internal standard. Mass spectra were obtained on a VG 70-250S spectrometer by a direct inlet system (Micromass Corp., Manchester, UK).

### 3.2. Plant Material

Whole *Cephalantheropsis gracilis* plants were collected from Pingtung Hsien, Taiwan, in December 2004, as authenticated by Chang-Sheng Kuoh, Department of Biology, National Cheng Kung University, Tainan, Taiwan. A voucher specimen (No: PLW-0401) was deposited in the Herbarium of National Cheng Kung University, Tainan, Taiwan.

### 3.3. Extraction and Isolation

The dried aerial parts of *C. gracilis* (2.4 kg) were extracted with MeOH (8 L) under reflux 8 times. The combined extracts were concentrated under reduced pressure to produce a dark brown syrup. The syrup was then suspended in H_2_O and then partitioned with hexane, CHCl_3_, and EtOAc, successively. The concentrated hexane layer (47 g) was chromatographed on a silica gel column by eluting with a gradient of hexane-Me_2_CO (10:1 to pure Me_2_CO) to give six fractions. Fraction 3 was chromatographed on silica gel by elution with hexane-*i*-Pr_2_O (1:3 to pure *i*-Pr_2_O) to give **48** (9.6 mg). Fraction 4 was chromatographed on silica gel using the same solvent mixture to yield **3** (4.3 mg), **34** (7.0 mg), **52** (0.7 mg), and **57** (1.4 mg). Fraction 5 was chromatographed on silica gel eluting with *i*-Pr_2_O (pure *i*-Pr_2_O to 30:1 of *i*-Pr_2_O-Me_2_CO to pure Me_2_CO) to give **4** (6.6 mg), **32** (3.5 mg), and **29** (5 mg).

The CHCl_3_ extract (30 g) was chromatographed on a silica gel column by eluting with a gradient of hexane-Me_2_CO (1:2 to pure Me_2_CO) to yield twelve fractions. Fraction 1 was subjected to chromatography on a silica gel column eluting with a gradient of hexane-Me_2_CO (10:1 to pure Me_2_CO) to give **10** (61.8 mg) and **39** (2.2 mg). Fraction 2 was chromatographed on a silica gel column eluting with a gradient of hexane-Me_2_CO (6:1 to pure Me_2_CO) to give **7** (39.4 mg), **2** (3.6 mg), **6** (543.1 mg), **13** (8.6 mg), and **17** (5.8 mg). Similarly, fraction 3 was chromatographed with a gradient of hexane-Me_2_CO (4:1 to pure Me_2_CO) to give **51** (2.2 mg) and **56** (22.7 mg). Fraction 5 was subjected to chromatography over silica gel eluting with a gradient of hexane-*i*-Pr_2_O (1:4 to pure *i*-Pr_2_O) to give **14** (20.0 mg) and **24** (48.0 mg). Fraction 6 was further purifed on a silica gel column eluting with a gradient of *i*-Pr_2_O-MeOH (50:1 to pure MeOH) to give **9** (34.4 mg), **19** (30.0 mg), and **28** (12 mg). Fraction 7 was chromatographed on a silica gel column eluting with a gradient of hexane-CHCl_3_ (6:1 to pure CHCl_3_) to give **25** (62.1 mg), **33** (0.7 mg), **5** (2.9 mg), **43** (11.4 mg), **44** (8 mg), and **45** (1.6 mg). Fraction 8 was subjected to chromatography over silica gel eluting with a gradient of CHCl_3_-Me_2_CO (30:1 to pure Me_2_CO) to give **12** (50.4 mg), **26** (2.7 mg), **31** (39.5 mg), **40** (3.4 mg), and **50** (8.3 mg). Fraction 9 was further chromatographed on a silica gel column eluting with a gradient of hexane-EtOAc (15:1 to pure EtOAc) to give **11** (54.1 mg) and **18** (6.2 mg). Fraction 10 was chromatographed on a silica gel column eluting with a gradient of *i*-Pr_2_O-MeOH (9:1 to pure MeOH) to give **27** (2.2 mg), **35** (2.3 mg), **42** (103 mg), **46** (1.2 mg), **49** (33.3 mg), and **37** (5.1 mg). Finally, fraction 11 was chromatographed on a silica gel column eluting with a gradient of CHCl_3_-MeOH (10:1 to pure MeOH) to give **53** (2.1 mg), **54** (37.4 mg), **55** (5.4 mg), and Fraction 12 yielded **23** (328.2 mg) as a pure crystalline material.

The EtOAc extract (20 g) was subjected to column chromatography using Cosmosil 75 C18 and eluted with a gradient of H_2_O-MeOH (from pure H_2_O to pure MeOH) to give nine fractions. Fraction 2 was subjected to further chromatography on a Cosmosil 75 C18 column eluting with a gradient of H_2_O–MeOH (from pure H_2_O to pure MeOH) to give **21** (3.6 mg) and **47** (12.5 mg). Fraction 3 was chromatographed on a silica gel column eluting with a gradient of *i*-Pr_2_O-MeOH (15:1 to pure MeOH) to give **22** (2.2 mg). Fraction 4 was subjected to repeated chromatography on a silica gel column eluting with a gradient of *i*-Pr_2_O–MeOH (10:1 to pure MeOH) to give **1** (1.4 mg), **16** (3.5 mg), **36** (12.6 mg), and **41** (44.5 mg). Fraction 5 was chromatographed on a silica gel column eluting with a gradient of *i*-Pr_2_O–MeOH (20:1 to pure MeOH) to give **8** (9.8 mg), **15** (22.2 mg), and **38** (2.9 mg). Fraction 6 was further purified over silica gel eluting with a gradient of CHCl_3_–MeOH (15:1 to pure MeOH) to give **20** (20.9 mg). Fraction 7 was further chromatographed on a silica gel column eluting with a gradient of *i*-Pr_2_O–MeOH (8:1 to pure MeOH) to give **30** (4.0 mg).

**Cephalanthrin**-**A** (**1**). White amorphous powder, mp 212–214 °C; [α]_D_ +8.0° (c 0.07, CH_3_OH); UV λ_max_ (log ε) CH_3_OH 206 (4.0), 261 (3.3), 302 (3.0), 314 (3.1), 329 (3.0) nm; IR ν_max_ (KBr) 3000 (br), 1652, 1464 cm^−1^; EIMS* m*/*z* (rel. int.) 308 (M^+^, 13), 250 (100), 219 (8), 119 (19); HREIMS *m*/*z* 308.0794 [M]^+^ (Calcd for C_17_H_12_N_2_O_4_, 308.0797).

**Cephalanthrin**-**B** (**2**). Yellow amorphous powder, mp 215–217 °C; [α]_D_ +3.0° (c 0.11, CHCl_3_); UV λ_max_ (log ε) CHCl_3_ 259 (3.2), 276 (3.0), 316 (2.9), 442 (2.9) nm; IR ν_max_ (KBr) 1754, 1722, 1643, 1607, 1592 cm^−1^; EIMS *m*/*z* (rel. int.) 322 (M^+^, 2), 291 (5), 263 (100); HREIMS *m*/*z* 322.0955 [M]^+^ (Calcd For C_18_H_14_N_2_O_4_, 322.0953).

**Cephathrene**-**A** (**3**). White amorphous powder, mp 96–98 °C; UV λ_max_ (log ε) CHCl_3_ 267 (3.3), 304 (3.2) nm; IR ν_max_ (KBr) 3404, 1582, 1483, 1467 cm^−1^; EIMS *m*/*z* (rel. int.) 302 (M^+^, 100), 255 (35), 184 (10); HREIMS *m*/*z* 302.1153 [M]^+^ (Calcd for C_17_H_18_O_5_, 302.1154).

**Cephathrene**-**B** (**4**). White amorphous powder; UV λ_max_ (log ε) CHCl_3_: 265 (3.2), 316 (3.0) nm; IR ν_max_ (KBr) 3400, 1606, 1501, 1464 cm^−1^; EIMS *m*/*z* (rel. int.) 332 (M^+^, 20), 331 (100), 302 (24), 285 (17); HREIMS *m*/*z* 332.1262 [M]^+^ (Calcd For C_18_H_20_O_6_, 332.1260).

**Methyl 2-(Aminocarbonyl)phenylcarbamate** (**5**). White amorphous powder, mp 200–202 °C; UV λ_max_ (log ε) CH_3_OH 212 (2.9), 228 (3.0), 257 (2.9), 289 (2.7), 311 (2.7) nm; IR ν_max_ (KBr) 3417, 3211, 1726, 1686, 1626, 1598, 1531 cm^−1^; ^1^H NMR (acetone-*d*_6_) δ 3.71 (3H, s, OCH_3_), 6.99 and 7.74 (each 1H, br s, NH_2_), 7.05 (1H, t, *J* = 7.8 Hz, H-4), 7.49 (1H, t, *J* = 7.8 Hz, H-5), 7.83 (1H, d, *J* = 7.8 Hz, H-3), 8.38 (1H, d, *J* = 7.8 Hz, H-6), 11.29 (1H, br s, 1-NH); ^13^C NMR (acetone-*d*_6_) δ 52.2 (OCH_3_), 119.2 (C-2), 119.6 (C-6), 122.2 (C-4), 129.1 (C-3), 133.4 (C-5), 141.7 (C-1), 154.6 (NC=O), 172.0 (2-C=O); EIMS *m*/*z* (rel. int.) 194 (M^+^, 31), 162 (32), 146 (100), 118 (9); HREIMS *m*/*z* 194.0693 [M]^+^ (calcd for C_9_H_10_N_2_O_3_, 194.0692).

### 3.4. Cytotoxicity Assay

The cytotoxicity assay was carried out according to the procedure described in the literature [17].

## 4. Conclusions

Five new compounds, cephalanthrin-A (**1**), cephalanthrin-B (**2**), cephathrene-A (**3**), cephathrene-B (**4**), methyl 2-(aminocarbonyl)phenylcarbamate (**5**), and 52 known compounds were isolated from *Cephalantheropsis gracilis*. Cephalinone D (**19**) showed the strongest cytotoxicity against the tested tumor cell lines, with IC_50_ values ranging from 7.6 to 42.9 μM. The modifications using Cephalinone D as template are being studied in our laboratories, aiming to discover the derivatives with strong anticancer activity.

## Supplementary Materials

Supplementary materials can be found at http://www.mdpi.com/1422-0067/16/02/3980/s1.
